# Current Insights of Post-Infusion CAR T Expansion and Persistence for Large B-Cell Lymphoma

**DOI:** 10.3390/cancers17193167

**Published:** 2025-09-29

**Authors:** Grace Wolyncewicz, Rebecca Wayte, Edward Abadir

**Affiliations:** 1 Institute of Haematology, Royal Prince Alfred Hospital, Sydney Local Health District, Sydney, NSW 2050, Australia; edward.abadir@health.nsw.gov.au; 2 Faculty of Medicine and Health, The University of Sydney, Sydney, NSW 2050, Australia; 3 Department of Haematology, Royal Adelaide Hospital, Adelaide, SA 5000, Australia; rebecca.wayte@sa.gov.au

**Keywords:** CAR T, lymphoma, large cell lymphoma, persistence, expansion, efficacy, toxicity

## Abstract

CD19 directed chimeric antigen receptor (CAR) T-cell therapy is standard of care for relapsed or refractory large B-cell lymphoma. CAR T-cell persistence and activity are associated with outcomes for patients with relapsed B-acute lymphoblastic leukaemia (B-ALL), but the association between expansion kinetics and outcome is less clear in the setting of large B-cell lymphoma. CAR T-cell expansion and persistence have been measured in both clinical trials and real-world settings, but the clinical relevance and applicability of these measurements remain unclear. There is increasing evidence that the in vivo kinetics of CAR T-cells post-infusion do offer important predictive insights into patient outcomes; despite this, limitations remain given the heterogeneity in methodology and timing of measurement. This review will summarise methodologies utilised to measure CD19 directed CAR T-cell expansion and persistence in vivo, in addition to the clinical implications of these measurements as currently described.

## Simple Summary

CD19 directed chimeric antigen receptor (CAR) T-cell therapy is standard of care for relapsed or refractory large B-cell lymphoma. CAR T expansion and persistence have been measured in multiple settings, but the clinical relevance of these measurements remain unclear. The aim of our review was to summarise current methods used to measure CAR T expansion and persistence, compare methods used between different studies, and assess whether there are any reported correlations between CAR T kinetics, efficacy, and toxicity.

## 1. Introduction

### Role of CAR T in Aggressive B-Cell Lymphoma

CD19 directed chimeric antigen receptor (CAR) T-cell therapy is standard of care for relapsed or refractory large B-cell non-Hodgkin lymphoma (LBCL). Prior to the advent of CAR T therapy, standard second line therapy for relapsed or refractory LBCL (R/R LBCL) in fit patients was salvage chemotherapy followed by autologous stem cell transplantation (ASCT), with 5 year event free survival rates of approximately 45% in those who respond to salvage and ASCT [1,2]. In patients who are less fit or those who relapse after autologous transplant, salvage chemotherapy was previously the only therapeutic option, with poor long-term survival and prognosis measured in months [3]. Outcomes for refractory or relapsed aggressive B-cell lymphoma have improved dramatically, with the advent of CAR T therapy, and multiple CD19-targeting products are now available. All CARs have a similar design incorporating a single chain variable fragment (scFv) that binds to the target antigen, in this case CD10, a costimulatory domain (either CD28 or 4-1BB), and a T-cell activation domain [4].

Currently, there are three CAR T-cell products targeting CD19 commercially available for use in large B-cell lymphoma. Axicabtagene ciloleucel (axi-cel, Kite/Gilead),which utilises a CD28 costimulatory domain, and tisagenlecleucel (tisa-cel, Novartis) and lisocabtagene maraleucel (liso-cel, BMS), which utilise a 4-1BB costimulatory domain, have been approved for use by both the European Medicines Agency (EMA) and the United States Food and Drug Administration (US FDA) [5,6,7]. The registrational studies ZUMA1 (axi-cel [8]), JULIET (tisa-cel [9]), and TRANSCEND (liso-cel [10] led to the approval of CD19 directed CAR for treatment of R/R DLBCL in patients who had already received two or more lines of therapy.

The phase 3 trials ZUMA-7 (Axi-cel [11]), BELINDA (tisa-cel [12]), and TRANSFORM (Liso-cel [13]) evaluated CD19-directed CAR T compared with salvage therapy followed by ASCT in patients with relapsed/refractory DLBCL within 12 months of completing first line therapy. Axi-cel and Liso-cel were subsequently approved for this indication by the EMA and US FDA.

ZUMA1 was a phase 2, single arm trial utilising axi-cel in 101 patients with LBCL refractory to chemotherapy or relapsed within 12 months of autologous stem cell transplant (ASCT). The 5 year follow-up data have now been published [14], which demonstrated a sustained overall and disease specific survival with an objective response rate of 83%, with 58% achieving a complete response (CR). Median overall survival (OS) was 25.8 months and the estimated 5 year overall survival rate was 42.6%.

ZUMA7, the phase 3 trial evaluating the use of axi-cel in patients with relapsed or refractory LBCL within 12 months of first line therapy, showed that the overall response rate (ORR) and complete response (CR) were significantly better in the axi-cel arm (83% and 65%) compared with standard salvage therapy (ORR 50% and CR 32%).

JULIET was the pivotal phase 2 trial that led to the approval of tisa-cel for R/R LBCL in the third line setting [9]. Ninety-three patients who had received two or more lines of therapy received tisa-cel, and in the primary analysis, the best overall response rate was 52% with a complete response rate of 40%. The long-term follow-up showed durable response, particularly in those who achieved an early CR. In the 3-year analysis of JULIET, the median progression-free survival (PFS) and OS were not reached in patients who achieved a CR at 3 or 6 months.

However, the BELINDA study failed to show a difference utilising tisa-cel over standard salvage, with an ORR of 46% and CR of 28% in the tisa-cel group, and an ORR of 43% and CR of 28% in the standard of care cohort.

The TRANSCEND NHL 001 study examined liso-cel in a cohort of R/R LBCL patients with a median of three lines of prior therapy. Liso-cel resulted in an ORR of 73% with a CR rate of 53%. TRANSCEND also demonstrated durable remission after liso-cel with an estimated 2 year PFS of 40.6% and OS of 50.5%.

Subsequently, the phase 3 trial TRANSFORM study showed that ORR and CR were significantly greater when liso-cel was used in the second line (ORR 86%, CR 66%) compared with the standard of care (ORR 48%, CR 32%).

Ultimately, only axi-cel and liso-cel have been approved for use in R/R DLBCL in the second line. The discrepancy between the results from different products in competing trials is unexplained; however, of note, there were significant differences in trial design including the degree of bridging therapy allowed, different definitions of event free survival (EFS), and the potential for crossover [15].

Given the increasing importance of CD19-directed CAR T in large B-cell lymphoma, it is important to define the most appropriate methodologies to measure the expansion and persistence of the CAR T cells in vivo, and to assess the clinical relevance of doing so. Therefore, we performed a literature review on the topic and included all major published papers and major registry data on the subject. For more relevant literature concerning the integration of this manuscript, please refer to the Supplementary Materials.

## 2. Methodologies to Measure CAR T

CAR T-cell expansion and persistence can be assessed by multiple techniques including digital droplet polymerase chain reaction (ddPCR), flow cytometry (FC), and quantitative real-time PCR (qPCR) [16]. The optimal time point for assessing peak CAR T-cell expansion is unclear [17]. 

### 2.1. Flow Cytometry

Flow cytometry utilises antibodies that bind specifically to the chimeric antigen receptor to detect the presence of the specific CAR protein expressed on the surface of the cell, which can be used to both quantify the number of CAR T-cells and evaluate functionality [18]. Two general methods for the detection of CAR surface products exist and are described by Selim et al. [19]. The first is a single step approach using fluorophore labelled anti-idiotype antibodies directed against specific epitopes of the CAR proteins that are not expressed by native CD19. Therefore, this assay will not erroneously detect normal B cells. One example of this method utilises an anti-FMC63 antibody-fluorophore conjugate. FMC63 is a murine protein comprising the antigen recognition site of the majority of CD19 directed CAR constructs. Therefore, an anti-FMC63 antibody will only bind cells expressing the CAR construct, rather than any native B cells expressing human CD19.

The second commonly utilised method is a two-step approach that involves use of a modified target protein, for example, biotinylated CD19, which is recognised and bound by the CAR, followed by the use of a secondary molecule such as streptavidin that is labelled with a fluorophore. Labelled streptavidin detects the biotin, and visualisation of the fluorophore indicates detection of the bound target protein. In addition, there are a number of different CAR-staining agents that have different target sites and properties. A number of other flow cytometric methods and staining agents are described in Table 1.

**Table 1:** Comparison of methodologies for measuring CAR T kinetics.

Methodology		Trials Utilising Method	Methodology Description	Advantages	Disadvantages
Flow cytometry—different staining agents/ methods	CAR staining agents targeting IgG-like fragments—polyclonal anti-IgG antibodies, and protein L		Detect antibody-based CARs without involving antigen binding site. Protein L selectively binds to kappa LC of immunoglobulins and Fab fragments, but not to their Fc portion.	Relatively cheap.	Batch to batch variation; Cross reactivity with non-CAR IgG like proteins on cell surface—requires multiple washing steps; Incompatible with antibodies and many FcX blocking reagents during MFC; Cannot independently stain different CARs on a dual CAR expressing T cell; Cannot stain CARs with a synthetic scFv.
	Antigen-Fc	Peinelt et al. [20] Badbharan et al. [21]	Takes advantage of the CAR’s binding affinity for its target antigen. Examples: CD19-FC (binds to anti-CD19 (FMC63) HER2-Fc, PSCA-Fc.	Can be used to evaluate expression of each specific CAR in dual CAR T cells. Commercially available from many vendors, e.g., Miltenyi Biotec ‘CD19 CAR Detection Reagent’.	More expensive; Possible decreased stability in solution; May be incompatible with FcX blocking reagents; The Fc fragment may non-specifically bind Fc receptors.
	Anti-idiotype antibodies	Abadir et al. [22] Hamilton et al. [22]	Specifically bind the variable regions of a particular scFv (e.g., against antiCD19 (FMC63) scFv). Example: ACRO biosystems antiFMC63 scFv.	High reagent stability. Low background staining. Compatibility with antibody panels in MFC. Can discriminate between different types of CARs. Can be used as a “cellular antidote” during therapy.	Can be difficult to obtain.
	Anti-linker antibodies	Used by Kite and Gilead sponsored studies involving axi-cel and tisa-cel	Rabbit monoclonal antibodies against 2 commonly used linkers in the CAR scFv that connect the heavy and light chains.		Not accessible to academic labs.
Genomic methods	ddPCR	Fehse [23], Badbaran [21]—utilised in patients receiving axi-cel and tisa-cel Ayuk et al. [24] TRANSCEND	Duplex ddPCR assay—concomitantly probed for the anti-CD19 CAR (FMC63 scFv) and a reference gene.	Excellent sensitivity and specificity. Limit of detection one single CAR transfused cell—sensitivity 0.01–0.02%—allows measurement of VCN in single cells. Good correlation with flow cytometry results.	ddPCR compatible technology less available and reactions more costly to run.
	qPCR	JULIET [8] ZUMA 19 February 2025 2:19:00 PM [8] ZUMA7	Primer and probe set used that were specific for the anti-CD19 CAR.		
		Wang et al. [25]	Primers for vector are based on FMC63 scFV sequence, and compared with reference gene (singleplex setup where vector and reference gene are amplified separately).	Robust across replicates. Minimum detection limit of 10 CAR copies per µL of blood.	Singleplex design—increased sample and reagent use, decreased throughput, increased pipetting noise.
		Kunz et al. [26]	Multiplex qPCR—FMC63 scFv and RNaseP (Rthe control) simultaneously qPCR amplified from the same gDNA sample using 2 independent fluorescent probes.	Similar efficiency as singleplex setup. Rapid and easily performed.	qPCR in general is not able to measure VCN at the single cell level.
		ELIANA, ENSIGN (B-ALL studies)—Mueller et al. [27,28]	qPCR (no further detail).		
		Peinelt et al. [20]	Developed primers for a unique region after sequencing the axi-cel CAR from FMC63 IGHV to TCR.		

The advantages of using flow cytometry include that this method is more readily available at the point of treatment than molecular methods, and the test can be performed rapidly to gain a result for an individual patient rather than waiting for batched results. In addition, it will only detect functional CAR expressed on the cell surface, meaning that flow cytometry will only enumerate CAR T-cells with functional antigen receptors. The disadvantages of flow cytometric methods are that the assays must be run within a short time frame after collection due to sample degradation, and flow is also less sensitive than some molecular methods, particularly ddPCR, as it has a sensitivity of approximately 0.1% [29].

### 2.2. Genomic Methods

During the CAR manufacturing process, T cells are virally transduced with a CAR vector that semi-randomly integrates into the T-cell genome [18]. Both digital droplet PCR (ddPCR) and qPCR quantitatively detect the integrated CAR transgene using probes specific to genomic DNA (gDNA) [18,30]. These methods do not demonstrate CAR surface expression or function, unlike flow cytometry [19].

### 2.3. Quantitative PCR (qPCR)

qPCR measures the frequency of the integrated CAR vector in the genome [18]. CAR-specific primers and fluorescent probes have been developed for both tisa-cel and axi-cel [21,23], which then use PCR to amplify the baseline level of DNA and quantitate the mean vector copy number (VCN—average vector copies per genome) using fluorescence [18]. The most common method of performing qPCR utilises primers to detect the anti-CD19 portion of the CAR construct (the tumour-associated antigen (TAA) binding region), which is based on the FMC63 scFV sequence [18,25]. Quantitative PCR assays with well-designed primers have been shown to be robust with good sensitivity (approximately 1%) [29]. These assays utilise technology that is frequently available in the clinical setting and can be used to measure CAR vector delivery efficiency, expansion kinetics, and persistence [18]. Limitations of qPCR include an inability to detect VCN at the single cell level and an inability to detect cell surface expression of the CAR. Surface CAR expression depends on other factors including regulatory elements of transcription and may mean that qPCR overestimates the true number of functional CAR T cells [18]. Additionally, the accuracy of qPCR depends on specific primers and probes.

### 2.4. Digital Droplet PCR (ddPCR)

Digital droplet PCR is a PCR technique that quantifies the copy number of CAR transgenes (VCN) independent of an external reference [30]. It does this by partitioning the entire gDNA sample into large numbers of individual PCR reactions contained in small droplets. Each individual reaction either contains the PCR target sequence or does not contain it, which is detected by fluorescence after PCR amplification is performed. The proportion of fluorescent droplets is then used to calculate the copy number [18]. ddPCR has been utilised in the clinical context to detect the CD19-targeting single chain fragment FMC63, which forms part of the tisa-cel, axi-cel, and liso-cel vectors. ddPCR has increased sensitivity and specificity for the detection of rare events compared with conventional PCR, and it can measure VCN in single cells, with sensitivity estimated at 0.01% [21,23,29]. This allows for an assessment of transduction efficacy and comparison from cell to cell [18]. The disadvantages of ddPCR include the increased running costs and the limited availability of the required technology.

### 2.5. Other Techniques

CAR T can also be detected at the genomic level by integration site analysis, which assesses the presence and genomic location of the integrated CAR vector [18]. Integration analysis uses NGS to find sites of “insertional mutagenesis”, where the CAR vector has been randomly inserted into the genome. Integration site analysis is useful in the setting of better understanding CAR T quality control and safety, for example, assessing where integration of a CAR may potentially be disrupting or lead to a CAR associated malignancy.

CAR detection at the transcriptomic level is possible by the detection of CAR mRNA, which is produced after the CAR integrates into the genome [18]. RNA-sequencing (RNA-Seq) is used to measure CAR mRNA abundance, and can be utilised at the single cell level so can identify which T cells have actually been transduced with the CAR. Single cell sequencing technologies can be used to identify the composition of T-cell subtypes, for example, CAR T cells with less differentiated naive and early memory features, which have been shown to be related to a higher rate of remission [31].

Cytometry by time-of-flight (CyTOF) is another single-cell technology combining both flow cytometry and mass spectrometry to detect protein expression at the single cell level [31]. This allows for high throughput analysis of the functional changes occurring in CAR T cells throughout the treatment period including activation and exhaustion [32].

#### Current State of Post-Infusion CAR T Expansion

A review of the current published literature found a trend towards higher peak CAR T expansion correlating with efficacy, and with more severe CRS. However, overall, there was still significant heterogeneity regarding the reported predictive insights provided by the measurement of the in vivo CAR T cell kinetics.

### 2.6. Timing and Method of Measurement

It is important to note that there was significant heterogeneity between the methodology and timing of measurements. As reported above, some publications utilised flow cytometry as a method of measuring CAR T expansion and persistence, and others utilised PCR, with differing sensitivities. A clear example of technique influencing the perception of CAR T persistence can be seen in JULIET, where there was a significant difference in measured CAR T persistence between flow cytometry and the qPCR-based methods. Maximum CAR T persistence was detected at 400 days when flow cytometry was used, but still detectable using qPCR at 693 days [7].

Inconsistencies were also present in the timing of measurements, with some trials measuring CAR T expansion more frequently than others. The day of maximal CAR T expansion consistently occurred in the first 14 days post-infusion, but there was a wide variation between trials with a range of 6–14 days reported. Where both flow cytometry and PCR were used, the day of maximal expansion was in some cases different between the two methods [7].

### 2.7. Peak Expansion and Efficacy

There were mixed conclusions across studies as to whether peak expansion and exposure correlated with patient outcomes in terms of efficacy. In the tisa-cel clinical trials, although the JULIET trial found a similar mean expansion in responders and non-responders, the BELINDA trial found that peak expansion was twice as high in patients who had a response than in those who did not respond to CAR T, and longer EFS in patients with a higher than median peak expansion [9,12]. In the clinical trials involving axi-cel, the ZUMA1 trial found a statistically significant association between higher expansion and response in patients treated with axi-cel, but the ZUMA7 trial did not find any association [8,11]. The TRANSCEND study found that the peak expansion of liso-cel CAR T cells was not correlated with overall survival, and TRANSFORM did not attempt to find a correlation between liso-cel expansion and outcome [5,13]. Hamilton et al. looked at 188 patients of whom the majority had DLBCL, and using flow cytometry, they did not find any association between peak expansion and patient response [22].

Several studies have attempted to define a threshold measurement for “low” expanders and “high” expanders, which best correlated with survival. For example, in the paper by Abadir et al., the “high” CAR T group was defined as >30 cells/µL as measured using flow cytometry, and they found that this high CAR T group was associated with a statistically lower risk of progression or death [16]. Blumenberg et al. found, also using flow cytometry, that >19 cells/µL was the threshold discriminating between responders and non-responders [17]. Ayuk et al. used ddPCR to find the median maximal expansion of 16.14 CAR T cells/µL, and deemed patients below this “weak” responders [24]. Although they only looked at 21 patients who received axi-cel, they found that all 10 “weak expanders” experienced progression—either requiring other lymphoma therapy or resulting in death. Compared with this, out of 11 patients defined as “strong” expanders with a peak expansion of 16.15 CAR T cells/µL or above, 9 were alive at median follow-up of 121 days including 8 without progression. Additionally, they found that PFS at 1 year was significantly higher for strong compared with weak expanders. Fehse et al. also found similar results using ddPCR, with a median peak expansion value of 11.2 CAR T cells/µL and a better 30 day clinical response for patients with peak expansion above this median [23]. ZUMA1 found that the median peak CAR T level using qPCR was higher in patients with an ongoing response at month 60 after infusion than in those who relapsed or did not have a response, but the median value itself was 65.76 cells/µL, so substantially higher than any of the values in those studies using ddPCR [8].

Overall, there appears to be a correlation between the response and expansion of CAR T cells to a level between 19 and 30 cells/µL as measured by flow cytometry, with the threshold less clear with the PCR-based measurement. Further work will be needed to prospectively validate these values.

### 2.8. Peak Expansion and Toxicity

The relationship between peak CAR T expansion and toxicity was more consistent, although the presence or absence of any correlations was not commented on in some studies. Almost all of the literature reviewed suggested a correlation between higher Cmax and/or AUC, and higher grades of CRS [14,16,22,24]. JULIET found that higher peak expansion and higher AUC correlated with Grade 3 or 4 CRS [33]. Abadir et al. found that the mean peak expansion was higher in patients with Grade 2 to 4 CRS (54.9 cells/µL in patients with Grade 2 to 4 CRS compared with 25.5 cells/µL in patients with Grade 0 to 1 CRS, *p* = 0.01), but there was no significant difference in peak expansion between those with ICANS and those without [16]. Ayuk et al. found a similar correlation between peak expansion being higher in patients with Grade 2 to 4 CRS compared with patients with Grade 0 to Grade 1 CRS (23.2 cells/µL versus 5.8 cells/µL, *p* = 0.07), but they also found that the peak expansion was higher in patients that experienced ICANS compared with those who did not [24] (25.0 cells/µL vs. 6.3 cells/µL, *p* = 0.09).

Conversely, ZUMA1 showed that peak expansion and AUC were significantly associated with neurological events but not with CRS [8]. They described the “peak factor change” for patients with neurologic events of Grade 3 or higher compared with Grade 0 to 2, and those with CRS of Grade 3 or higher compared with 0 to 2. The peak factor was 2.1 for neurologic events, showing an association, but only 1.1 for CRS [8]. Wittschlager et al. similarly did not find any correlation with CAR T persistence at 6 months and the presence of prior CRS in their patients, but did find that CAR T persistence at 6 months was associated with a higher incidence of ICANS, with only 7% of patients who were ddPCR-negative having experienced ICANS previously, whilst 37% of those who were ddPCR-positive had [34].

### 2.9. Persistence and Efficacy

Regarding CAR T persistence and any difference between products, some mouse models as well as clinical trials had previously demonstrated longer persistence in products utilising a 4-1BB compared with those utilising a CD28 costimulatory domain in some studies [4,7,10]. However this may be influenced by the differences in methodology used to measure persistence and the sensitivity of the assays, and as noted in Table 2, multiple trials have now shown long-term CAR T persistence of more than a year with the use of a product with either costimulatory domain [9,10,14,35,36].

**Table 2:** CAR T expansion, persistence, and exposure, and correlations to efficacy and toxicity.

Trial	Population and Methods Used	Peak Expansion Value (Cmax) and Timing (Tmax)	CAR T Persistence (Tlast)	Efficacy Correlation	Toxicity Correlation
JULIET [9,35,37]	Phase 2 Trial of tisa-cel in R/R DLBCL. FC (%CD3 + CAR + cells). qPCR (transgene copies/µg).	Cmax and Tmax FC Responders: =4.81% at 6.35 days. Non responders: =4.18% at 7.64 days. Cmax and Tmax qPCR: Responders: 6470 at 9.8 days. Non responders: 5050 at 9 days.	FC Responders: median 280 days. Maximum 554 days. Non responders: median 28 days. Maximum 400 days. qPCR: Responders: Median 180 days. Maximum 693 days. Non responders: Median 59 days.	qPCR Similar Cmax, tmax in responders and non-responders. Higher than median Cmax = potentially longer DOR (not statistically significant). Persistent CAR transgene levels up to 2 years in patients with ongoing response.	Higher Cmax and AUC associated with Grade 3 or 4 CRS (OR with 2-fold increase in Cmax, 1.70). No relationship with ICANS.
BELINDA [12]	Phase 3 Trial. Tisa-cel in 2nd line treatment of DLBCL. qPCR.	No specific numbers given.	Transgene detectable in 53/54 at 4 months.	Cmax twice as high in responders as non-responders. Similar Tmax. Longer EFS if higher than median Cmax. 18/38 had quantifiable CAR transgene at relapse.	
ZUMA 1 [8,14]	Phase 2. Axi-cel in R/R DLBCL. qPCR.	Tmax = 14 days. Cmax: Responders: 65.76 cells/µL. Relapse: 35.27 cells/µL. No response: 12.08 cells/µL.	CAR T cells detectable in most patients at 180 days. 3 patients in CR at 24 months with detectable CAR T.	CMax significantly associated with response (*p* < 0.001). Exposure associated with response. Ongoing response at 60 months associated with higher early CAR T-cell expansion and higher AUC.	Peak expansion and AUC significantly associated with neurologic events, but not with CRS
Locke et al. —ZUMA1 data [38]	Analysed biomarker data from ZUMA1. qPCR. ddPCR. Normalised Cmax to tumour burden (TB).	No specific numbers given.	No specific numbers given. CAR T cells >= 3 months low or non-measurable.	Higher CMax and AUC0-28 days associated with response. Strongest correlate of durable response was Cmax normalised to pretreatment TB.	Higher Cmax and baseline TB associated with grade ≥3 ICANS but not grade ≥3 CRS.
ZUMA 7 [6,11]	Phase 3. Axi-cel in 2nd line. qPCR.	Median Cmax 25.84 cells per cubic/mL. Median Tmax CAR T was 7 days.	CAR T cells detectable in 12 of 30 patients at 24 months.	Cmax and AUC0-28 were not significantly associated with OS.	
TRANSCEND NHL 001 [5]	Phase 2. Liso-cel in R/R DLBCL. qPCR used until month 24. ddPCR from month 30.		Persistence rate 37% at 24 months, and 43% at 42 months.	Cmax and AUC not associated with OS.	
TRANSFORM [13,39]	Phase 3. Liso-cel in 2nd line. ddPCR.	Median Cmax 33 285 copies per µg. Median Tmax 10 days.	Persistence seen up to 23 months after infusion.		
Abadir/Wayte [16]	Axi-cel or tisa-cel, after 2 or more lines of therapy. FC detecting FMC63.	Mean expansion 64.7 cells/µL. Responders = 80.3 cells/µL. Non-responders = 26.4 cells/µL. “High CAR T group” delineated as >30 cells/µL.		Cmax greater in responders. “High CAR T group” (>30 cells/µL) lower risk of progression or death. No difference in response rates b/w products.	Cmax higher in patients with G2–4 CRS. No association with ICANS.
Ayuk et al. [24]	21 patients receiving axi-cel for DLBCL or PMBCL—median 5 lines prior therapy. ddPCR.	Median Cmax = 16.14 CAR T cells/µL. (Below = “weak” responders).	Persistence in 8/10 at 6 months, with median of 0.50 CAR T cells/µL. Persistence in 3/7 patients in CR at 12 months.	At median follow up: 10/10 “weak expanders” progressed needing treatment, or died. 9/11 strong expanders alive, 8 in CR, PR or SD. PFS at 1 year: 71% vs. 0% for strong and weak expanders (*p* < 0.001). Cmax higher in patients with CR or PR compared with non-responders (22.06 vs. 3.02 cells per µ/L, *p* = 0.006).	CMax higher for patients with CRS G2-4 compared with G0 to 1 (23.2 vs. 5.8 per u/L, *p* = 0.07). Cmax higher for patients with ICANS (25 vs. 6.3 per µL, *p* = 0.09).
Demaret [40]	28 patients receiving axi-cel in French university hospital. Flow cytometry using labelled CD19 protein.	Tmax 12 days after infusion.		No clinical correlation performed due to small numbers.	
Blumenberg [17]	Patients receiving third line tisa-cel or axi-cel. FC—biotinylated recombinant CD19 protein.	D +7 expansion most significant. 19 cells/µL identified value for discriminating between responders and non-responders.		Association between D +7 CAR levels and D +30 response and survival. ECOG, ferritin at baseline negatively associated with CAR T levels on D +7.	
Hamilton [22]	188 lymphoma patients treated with CD19 directed CAR—majority DLBCL. FC—anti-idiotype antibody directed against FMC63.	Expansion measured weekly. Median percent CAR at D +7 was 14.67% of T cells, and 1.33% at D +28. Tmax D +14.		Younger patients, and elevated LDH had significantly higher Cmax. No association between Cmax and PFS. No association between Cmax and best clinical response. No difference in Cmax in patients with or without progression.	AUC significantly associated with CRS grade (*p* < 0.0001), ICANS grade (*p* < 0.001%). CD4:8 ratio signif lower in patients with severe ICANS compared with less severe or no ICANS (*p* < 0.001).
Wittischlager [34]	92 patients with R/R B-cell lymphoma (majority DLBCL) who received CD19 targeted CAR T. ddPCR.	Cmax 4859.5 copies/µg. Tmax 9 days. Median Cmax levels were similar between products.	Median time to undetectable CAR T 98 days. At month +6, persistence detected in 84%. At month +6 tisa-cel persistence = 96%, axi-cel persistence 73%.	CAR T persistence at 6 months assoc with higher Cmax (5432 vs. 620 copies/µg in persistent pts vs. non persistent). Relapse less frequent in patients with persistence (29% vs. 60%). Persistence at 6 mo assoc with longer PFS, and trend towards improved OS. No significant differences in CR or PR rates between patients with vs. without CAR T persistence.	CRS and high grade CRS frequency not associated with persistence. CAR T persistence at 6 months assoc with higher incidence of ICANS (37% vs. 7%).
Fehse [23]	16 patients who received axi-cel. ddPCR.	Median Cmax 11.2/µL.		Trend for association between Cmax above median and better D +30 clinical response (CR, PR, SD).	

Abbreviations: CR = complete response, PR = partial response, SD = stable disease, Responders = CR or PR. Non-responders = SD, unknown. Cmax Maximal expansion of transgene/CAR-positive T-cell levels in vivo post-infusion. Tmax: time to maximal expansion. AUC: exposure up to 28 days (area under the curve [AUC]0–28D). Tlast: persistence (duration transgene/CAR-T cells are present in peripheral blood and tissues. DOR: duration of response.

There was significant variability in the length of CAR T persistence between trials, and even within trials where multiple methods of measuring CAR T presence were used, as above. Several studies did suggest a correlation between CAR T persistence and response. Median CAR T persistence was longer in responders compared with non-responders when measured by both FC and qPCR in JULIET [7]. Wittischlager et al. [34] found that CAR T persistence at 6 months occurred in 84% of their patients, and persistence was associated with a higher initial CAR T peak. Although there were no significant differences in the specific response criteria met (e.g., CR, PR) between patients with detectable CAR at 6 months compared with those without, in the group with persistence, relapse was less common and there was an association with longer PFS and a trend towards improved OS.

However, the significance of ongoing CAR T persistence in LBCL is debatable, as several trials found detectable CAR transgene levels, even in patients who had relapsed after initially responding to CAR T [12]. The two studies utilising liso-cel observed persistence of the transgene up to 24 months post-infusion, but did not comment on any correlation to response. ZUMA1 and ZUMA7 did not provide any data regarding correlation of response to persistence of CAR T.

## 3. Discussion

Overall, the literature reviewed showed significant heterogeneity between timing and methods of measuring CAR T expansion and persistence, and the reporting of results was widely disparate. While there is a suggestion that there is a likely correlation between CAR T expansion and response in patients with DLBCL receiving CAR T, and a correlation between expansion and toxicity (particularly CRS), there is a need for the standardisation of measurement and clear protocols, if measurements of cellular kinetics are to be integrated into the day-to-day management of CAR T patients. It is also difficult to directly compare all of the studies above due to differences in the infused products.

Flow cytometry appears to be the most widely available method that could be utilised in real-time to assess CAR T expansion during the 6–14 day postulated range where peak expansion is thought to occur. Prospective validation is required; however, several studies have suggested a range that may correspond with “strong” and “weak” responders, and additionally, values that may correspond with higher toxicity. The utility of acquiring peak expansion measurements at this time point would be the identification of patients in whom early disease assessment should be prioritised, both with imaging and with circulating tumour (ct) DNA. The utility of using expansion to predict toxicity is less clear, as realistically, toxicity will generally be occurring at the same time as the measurement of expansion is being performed. The utility of measuring persistence remains unclear and needs to be explored further, as currently, there is no clear trend to indicate that early loss of CAR persistence in LBCL is a surrogate for poor response.

### 3.1. Comparison with ALL

In contrast to the disparate data in LBCL, CAR T persistence and the presence of B-cell aplasia has been consistently shown to correlate with response in B-ALL. Both qPCR and flow cytometry have been utilised in the registrational and randomised studies that contributed to the approval of tisa-cel in ALL [28,36]. Peak expansion generally occurred at 10–14 days, and patients with a complete response had higher peak expansion levels of tisa-cel than patients who did not respond (*p* < 0.0001). In some patients with prolonged persistence, tisa-cel was still detectable 2 years after infusion. In ALL, there was also a consistent association between CAR T expansion, tumour burden, and CRS—high grades of CRS were seen in patients with higher baseline tumour burden and higher expansion. [27].

### 3.2. Comparison with Alternatives and Future Directions

The measurement of tumour ctDNA is an alternative method for patient prognostication. Currently, most assays used to detect ctDNA are performed via next generation sequencing (NGS), and most commonly use high throughput sequencing to detect a unique marker of clonality in B-cell malignancies as a result of variable, diversity, and joining gene segment (VDJ) recombination in the immunoglobulin genes [41] to determine a tumour clonotype. Cancer personalised profiling by deep sequencing (CAPP-Seq) is another NGS method that utilises a panel including genomic regions with recurrent somatic alternations in DLBCL [42]. NGS uses probes to capture specific DNA sequences prior to sequencing and allows for the identification of multiple mutations even without prior knowledge of disease specific genomic alterations [43]. CtDNA can also be detected using PCR-based methods such as ddPCR. While this method is more readily available than NGS, it is limited to known mutations and cannot be used as broadly as NGS [43]. Several newer methods not yet in routine use include time-of-flight mass cytometry (CyTOF), which combines flow cytometry with mass spectrometry, and surface enhanced Raman spectroscopy (SERS) [43].

Measurement of tumour ctDNA using NGS has been employed at multiple clinically relevant time points in patients receiving CAR T, for example, measurement prior to CAR T infusion to identify a patient clonotype and assess the pretreatment ctDNA concentration [41], and then as an ongoing measure of disease response to therapy and therefore prognosis [19].

Frank et al. employed the Adaptive Clonoseq assay in patients who had received axi-cel. Higher ctDNA concentrations before receiving axi-cel were associated with progression after axi-cel infusion, and also associated with developing CRS and ICANS. A tumour clonotype was detectable in 69 out of 72 (96%) enrolled patients prior to infusion. At day +28, patients with detectable ctDNA compared with those with undetectable ctDNA had a median PFS and OS of 3 months vs. not reached (*p* < 0.0001), and 19 months vs. not reached (*p* = 0.0080), respectively. CtDNA was detected at or before PET relapse in 29/30 (94%) of patients, while conversely, all patients with a durable response had undetectable ctDNA at or before 3 months after receiving axi-cel [41].

The available literature suggests that early after CAR T therapy, peripheral blood ctDNA assessment can aid in predicting progression. Lymphoma specific clonotypes can be identified in the majority of patients, and these assays have good sensitivity (0.01%). However, the clinical sensitivity depends on the amount of “shedding” of ctDNA from the tumour [19]. The technology for performing ctDNA assessment is not widely available, is labour and time intensive, and turnaround time can be slow. The detection of mutations can also be difficult when the tumour burden is low. Given this, measuring the CAR T kinetics instead by using the assays described earlier in this review may be a more attractive and realistic option. The assays used to measure expansion kinetics, particularly flow cytometry-based assays, can give results within days and can be run at a local treatment centre where the result may enable the prediction of relapse or toxicity, and therefore earlier intervention or monitoring. In contrast, ctDNA potentially may not give results in a clinically meaningful timeframe, and may not be a valid marker in the very early time points post-infusion.

Given the clinical need to identify those patients at risk of CAR T failure and disease progression, the measurement of CAR T expansion may also be useful in conjunction with ctDNA measurement—for example, poor expansion could be used to identify patients in whom performing ctDNA measurement should be prioritised.

The ability to adopt other state-of-the-art methodology such as RNA-Seq and CyTOF into clinical practice is less clear, given the turnaround time and paucity of clinical facilities who have access to this technology. These methodologies should first be employed more widely in the research context to try and assess whether, for example, CAR T exhaustion seems to have any clinical impact on patient relapse, and if these tests can realistically be performed in a useful time frame to predict relapse.

## 4. Conclusions

Despite the improvement in outcomes in R/R LBCL in the CAR T-cell era, many patients still experience relapse. Increasing evidence suggests that the in vivo kinetics of CAR T-cells post-infusion offer important predictive insights into both CAR T efficacy and toxicity, and measurement using flow cytometry and genomic methods could be combined with other emerging technologies such as ctDNA. Limitations currently exist in the measurement of CAR T expansion, with a lack of standardisation of both the methodology and timing of measurement, and more work needs to be performed to prospectively validate the threshold of CAR T expansion that is clinically relevant for each product.

## Supplementary Materials

The following supporting information can be downloaded at: https://www.mdpi.com/article/10.3390/cancers17193167/s1, Supplementary file: Additional references: [26,44,45,46,47,48,49,50,51,52,53,54,55,56,57,58,59,60,61,62,63,64,65].
