# Supplementary material for: Current Insights of Post-Infusion CAR T Expansion and Persistence for Large B-Cell Lymphoma
Source: Cancers (Basel). 2025 Sep 29;17(19):3167. doi: 10.3390/cancers17193167 (PMC12523718; doi:10.3390/cancers17193167)
Supplement: Supplementary file 1 [file cancers-17-03167-s001.zip › cancers-3878762-supplementary.pdf]

Additional references

1. Kunz, A.; Gern, U.; Schmitt, A.; Neuber, B.; Wang, L.; Hückelhoven-Krauss, A.; Michels, B.; Hofmann, S.; Müller-Tidow, C.; Dreger, P.; et al. Optimized Assessment of qPCR-Based Vector Copy Numbers as a Safety Parameter for GMP-Grade CAR T Cells and Monitoring of Frequency in Patients. *Mol. Ther.—Methods Clin. Dev.* **2020**, *17*, 448–454. https://doi.org/10.1016/j.omtm.2020.02.003.
2. Haradhvala, N.J.; Leick, M.B.; Maurer, K.; Gohil, S.H.; Larson, R.C.; Yao, N.; Gallagher, K.M.E.; Katsis, K.; Frigault, M.J.; Southard, J.; et al. Distinct cellular dynamics associated with response to CAR-T therapy for refractory B cell lymphoma. *Nat. Med.* **2022**, *28*, 1848–1859. https://doi.org/10.1038/s41591-022-01959-0.
3. Dean, E.A.; Kimmel, G.J.; Frank, M.J.; Bukhari, A.; Hossain, N.M.; Jain, M.D.; Dahiya, S.; Miklos, D.B.; Altrock, P.M.; Locke, F.L. Circulating tumor DNA adds specificity to PET after axicabtagene ciloleucel in large B-cell lymphoma. *Blood Adv.* **2023**, *7*, 4608–4618. https://doi.org/10.1182/bloodadvances.2022009426.
4. Samara, J.A.; Baron, M.; Gazzano, M.; Parizot, C.; Kirupaharan, M.; Guihot, A.; Miyara, M.; Gorochov, G.; Choquet, S.; Sterlin, D. CD8 and CD4 CAR-T cells are associated with outcome and toxicity of tisagenlecleucel in central nervous system lymphoma. *Cytotherapy* **2025**, *27*, 933–937. https://doi.org/10.1016/j.jcyt.2025.05.005.
5. Cao, G.; Hu, Y.; Pan, T.; Tang, E.; Asby, N.; Althaus, T.; Wan, J.; Riedell, P.A.; Bishop, M.R.; Kline, J.P.; et al. Two-Stage CD8$^+$ CAR T-Cell Differentiation in Patients with Large B-Cell Lymphoma 2025. https://doi.org/10.1101/2025.03.05.641715.
6. Zhu, Y.; Xu, K.; Wang, Y. Tumor microenvironment in CAR-T cell therapy for lymphoma. *Best Pract. Res. Clin. Haematol.* **2025**, *38*, 101635. https://doi.org/10.1016/j.beha.2025.101635.
7. Xu, Y.; Zhang, M.; Ramos, C.A.; Durett, A.; Liu, E.; Dakhova, O.; Liu, H.; Creighton, C.J.; Gee, A.P.; Heslop, H.E.; et al. Closely related T-memory stem cells correlate with in vivo expansion of CAR.CD19-T cells and are preserved by IL-7 and IL-15. *Blood* **2014**, *123*, 3750–3759. https://doi.org/10.1182/blood-2014-01-552174.
8. Lionel, A.C.; Neelapu, S.S. CAR T-cell expansion: Harmful or helpful? *Blood Adv.* **2024**, *8*, 3311–3313. https://doi.org/10.1182/bloodadvances.2024013146.
9. Cappell, K.M.; Kochenderfer, J.N. Long-term outcomes following CAR T cell therapy: What we know so far. *Nat. Rev. Clin. Oncol.* **2023**, *20*, 359–371. https://doi.org/10.1038/s41571-023-00754-1.
10. Baur, K.; Buser, A.; Jeker, L.T.; Khanna, N.; Läubli, H.; Heim, D.; Dirks, J.C.; Widmer, C.C.; Volken, T.; Passweg, J.R.; et al. CD4+ CAR T-cell expansion is associated with response and therapy related toxicities in patients with B-cell lymphomas. *Bone Marrow Transplant.* **2023**, *58*, 1048–1050. https://doi.org/10.1038/s41409-023-02016-1.
11. Shiqi, L.; Jiasi, Z.; Lvzhe, C.; Huailong, X.; Liping, H.; Lin, L.; Qianzhen, Z.; Zhongtao, Y.; Junjie, S.; Zucong, C.; et al. Durable remission related to CAR-T persistence in R/R B-ALL and long-term persistence potential of prime CAR-T. *Mol. Ther.—Oncolytics* **2023**, *29*, 107–117. https://doi.org/10.1016/j.omto.2023.04.003.
12. Yamauchi, N.; Maruyama, D. Current development of chimeric antigen receptor T-cell therapy for diffuse large B-cell lymphoma and high-grade B-cell lymphoma. *Eur. J. Haematol.* **2024**, *112*, 662–677. https://doi.org/10.1111/ejh.14166.
13. Van Den Neste, E.; Schmitz, N.; Mounier, N.; Gill, D.; Linch, D.; Trneny, M.; Milpied, N.; Radford, J.; Ketterer, N.; Shpilberg, O.; et al. Outcome of patients with relapsed diffuse large B-cell lymphoma who fail second-line salvage regimens in the International CORAL study. *Bone Marrow Transplant.* **2016**, *51*, 51–57. https://doi.org/10.1038/bmt.2015.213.
14. Turicek, D.P.; Giordani, V.M.; Moraly, J.; Taylor, N.; Shah, N.N. CAR T-cell detection scoping review: An essential biomarker in critical need of standardization. *J. Immunother. Cancer* **2023**, *11*, e006596. https://doi.org/10.1136/jitc-2022-006596.
15. Maryamchik, E.; Gallagher, K.M.E.; Preffer, F.I.; Kadauke, S.; Maus, M.V. New directions in chimeric antigen receptor T cell [CAR-T] therapy and related flow cytometry. *Cytometry B Clin. Cytom.* **2020**, *98*, 299–327. https://doi.org/10.1002/cyto.b.21880.
16. Zheng, Z.; Chinnasamy, N.; Morgan, R.A. Protein L: A novel reagent for the detection of Chimeric Antigen Receptor (CAR) expression by flow cytometry. *J. Transl. Med.* **2012**, *10*, 29. https://doi.org/10.1186/1479-5876-10-29.
17. Sworder, B.J.; Kurtz, D.M.; Alig, S.K.; Frank, M.J.; Shukla, N.; Garofalo, A.; Macaulay, C.W.; Shahrokh Esfahani, M.; Olsen, M.N.; Hamilton, J.; et al. Determinants of resistance to engineered T cell therapies targeting CD19 in large B cell lymphomas. *Cancer Cell* **2023**, *41*, 210–225.e5. https://doi.org/10.1016/j.ccell.2022.12.005.

18. Good, Z.; Spiegel, J.Y.; Sahaf, B.; Malipatlolla, M.B.; Ehlinger, Z.J.; Kurra, S.; Desai, M.H.; Reynolds, W.D.; Wong Lin, A.; Vandris, P.; et al. Post-infusion CAR TReg cells identify patients resistant to CD19-CAR therapy. *Nat. Med.* **2022**, *28*, 1860–1871. https://doi.org/10.1038/s41591-022-01960-7.
19. Maude, S.L.; Laetsch, T.W.; Buechner, J.; Rives, S.; Boyer, M.; Bittencourt, H.; Bader, P.; Verneris, M.R.; Stefanski, H.E.; Myers, G.D.; et al. Tisagenlecleucel in Children and Young Adults with B-Cell Lymphoblastic Leukemia. *N. Engl. J. Med.* **2018**, *378*, 439–448. https://doi.org/10.1056/NEJMoa1709866.
20. Frank, M.J.; Hossain, N.; Bukhari, A.; Dean, E.; Spiegel, J.Y.; Claire, G.K.; Kirsch, I.M.; Jacob, A.P.; Mullins, C.D.; Lee, L.W.; et al. Detectable Circulating Tumor DNA 28 Days after the CD19 CAR T-Cell Therapy, Axicabtagene Ciloleucel, Is Associated with Poor Outcomes in Patients with Diffuse Large B-Cell Lymphoma. *Blood* **2019**, *134*, 884. https://doi.org/10.1182/blood-2019-132057.
21. Kurtz, D.M.; Scherer, F.; Jin, M.C.; Soo, J.; Craig, A.F.M.; Esfahani, M.S.; Chabon, J.J.; Stehr, H.; Liu, C.L.; Tibshirani, R.; et al. Circulating Tumor DNA Measurements As Early Outcome Predictors in Diffuse Large B-Cell Lymphoma. *J. Clin. Oncol.* **2018**, *36*, 2845–2853. https://doi.org/10.1200/JCO.2018.78.5246.
22. Schanda, N.; Sauer, T.; Kunz, A.; Hückelhoven-Krauss, A.; Neuber, B.; Wang, L.; Hinkelbein, M.; Sedloev, D.; He, B.; Schubert, M.-L.; et al. Sensitivity and Specificity of CD19.CAR-T Cell Detection by Flow Cytometry and PCR. *Cells* **2021**, *10*, 3208. https://doi.org/10.3390/cells10113208.
23. García-Calderón, C.B.; Sierro-Martínez, B.; García-Guerrero, E.; Sanoja-Flores, L.; Muñoz-García, R.; Ruiz-Maldonado, V.; Jimenez-Leon, M.R.; Delgado-Serrano, J.; Molinos-Quintana, Á.; Guijarro-Albaladejo, B.; et al. Monitoring of kinetics and exhaustion markers of circulating CAR-T cells as early predictive factors in patients with B-cell malignancies. *Front. Immunol.* 2023, *14*, 1152498. https://doi.org/10.3389/fimmu.2023.1152498.
